# GABA Signaling in Health and Disease in the Nervous System

**DOI:** 10.3390/ijms252011193

**Published:** 2024-10-17

**Authors:** Alexandre Hiroaki Kihara

**Affiliations:** 1 Neurogenetics Laboratory, Universidade Federal do ABC, São Bernardo do Campo 09606-045, SP, Brazil; alexandrekihara@gmail.com; 2 Center for Mathematics, Computing and Cognition, Universidade Federal do ABC, São Bernardo do Campo 09606-045, SP, Brazil

Throughout development, gamma-aminobutyric acid, or GABA, plays a role in the proliferation, migration, and differentiation of neural progenitor cells. The maturation of synapses and the process of neuritogenesis were both demonstrated to be controlled by GABA. Among the neurotransmitters that contribute to the excitatory-inhibitory balance in the mature brain, GABA is believed to be the primary inhibitory neurotransmitter. Neurodevelopmental disorders and neurodegenerative diseases are frequently linked to shifts in this equilibrium [1,2], which may correlate to decreasing inhibitory neurons. Indeed, recent reports have demonstrated that hippocampal GABAergic neurons play roles that were not previously assigned, such as coding space and speed [3], which may subserve cognition impairments observed in autism and Alzheimer’s disease. Besides the central nervous system, GABAergic neurons are also observed in the peripheral nervous system. All these topics were covered in this Special Issue and listed as contributions.

In the postnatal brain, the greatest proliferative cell group outside neurogenic niches is the NG2 glia. In white matter, most NG2 cells differentiate into oligodendrocytes, which is why they are also known as oligodendrocyte progenitor cells (OPCs). The functional effect of neuronal synaptic input on NG2 glia is unknown. Patt et al. evaluated spontaneous and evoked GABA_A_ receptor-mediated currents of NG2 glia in juvenile and adult hippocampi of mice and examined interneuron–glial signaling alterations over development. Both patch-clamp and pharmacological investigations showed reduced hippocampal NG2 glia innervation between postnatal days 10 and 60. Adults had increased extrasynaptic receptor activity. Switching from synaptic to extrasynaptic receptor stimulation led to γ2 downregulation and α5 subunit overexpression. High-resolution expansion microscopy and molecular studies revealed glial GABA_A_ receptor trafficking and clustering. Gephyrin and radixin cluster separately along glial pathways. Interestingly, deleting γ2 and postsynaptic receptors did not affect the glial production of scaffolding proteins, auxiliary receptor subunits, or interaction proteins. The authors conclude that GABAergic input to NG2 glia may regulate the release of neurotrophic substances that affect neuronal synaptic plasticity.

During brain development, serotonergic neurons are among the first cells to differentiate. Serotonin (5-HT) modulates activity-dependent synaptic alterations throughout the critical phase of the primary visual cortex (V1). Many studies have shown that pyramidal neurons in the superficial layers of V1’s synaptic plasticity depend on a delicate balance between excitation and inhibition (E/I). Carlos-Lima et al. used intracellular electrophysiological recordings and showed that 5-HT primarily reduces E/I balance throughout the critical period. The study revealed that 5-HT increased LTD at excitatory synapses and blocked it at inhibitory synapses, altering Hebbian synaptic strength toward lower E/I balance.

GABA_B_ receptor-mediated inhibition is essential for neuronal excitation/inhibition balance. Due to increased lysosomal degradation of GABA_B_ receptors, many neurological disorders are associated with a disrupted excitation/inhibition balance. A critical process in receptor downregulation is the CaMKIIβ-mediated phosphorylation of S867 in the GABA_B1_ subunit observed in ischemic conditions. Bhat et al. demonstrated that silencing another phosphorylation site, T872 at S867, inhibited receptor degradation in cultured neurons. The authors also observed that CaMKIIβ does not directly phosphorylate S867. Instead, observations indicated that CaMKIIβ stimulates ERK1/2, which phosphorylates S867 and T872 sites in GABA_B1_. Thus, inhibiting these sites can prevent pathogenic receptor downregulation after ischemia stress and restore neuronal excitation/inhibition balance.

In CA1 of the hippocampus, the indirect effects of oxytocin (OT) on pyramidal neurons and information processing have been identified. However, little is known about how OT directly modulates CA1 interneurons expressing the oxytocin receptor (OTR). Castagno et al. demonstrated that TGOT, a selective OTR agonist, changes the membrane potential, firing frequency, neuronal excitability, and action potential shape of mice CA1 interneurons. Using linear mixed-effects models, the authors showed that OT modulates CA1 GABAergic interneurons, driving independent alterations in the shape of action potentials and increasing the firing rate.

Several studies suggest that hippocampal hyperactivity is fundamental to the pathogenesis of both temporal lobe epilepsy (TLE) and psychosis; therefore, reducing hippocampal activity to normal levels may be a potential treatment for these conditions. In mouse models, increased ventral hippocampus (vHipp) activity causes dopamine system dysfunction, which may subserve psychotic symptoms. It was previously observed that targeting α5-containing GABA receptors (α5GABAARs) with positive-allosteric modulators (PAMs) in the hippocampus restores dopamine system function in hyperactive mouse models. The findings suggest that α5-PAMs may be useful in TLE treatment. McCoy et al. found that TLE leads to enhanced VTA dopamine cell activity. However, intra-vHipp injection of GL-II-73, a selective α5-PAM, completely reversed this effect. The authors proposed that enhancing α5GABAARs function may be a potential therapy for psychosis in TLE.

Alzheimer’s disease (AD) disproportionately affects women. AD symptoms include memory loss due to Aβ plaques and synapse breakdown. The extracellular matrix’s perineuronal net (PNN) stabilizes synapses and is impacted by microglia, which are activated during AD. PNN was described as a more condensed cartilage-like extracellular matrix surrounding neurons, particularly GABAergic parvalbumin-positive interneurons [4]. Using an amyloid precursor protein knock-in mouse model of AD, Rahmani et al. examined sex variations in PNN, microglia, and Aβ plaque density. The highest density of PNN was observed in the lateral entorhinal cortex and presubiculum regions of aged AD mice with region-specific sex differences. The study also revealed extensive Aβ plaques surrounding active microglia and PNN in the CA1 area. The authors proposed that sex-related variations alter PNN-microglia interaction in AD-related brain areas.

Finally, besides the brain, GABAergic neurons are also observed in the peripheral sympathetic nervous system. For instance, GABA and acetylcholine (ACh) can colocalize or be separated in superior cervical ganglia (SCG) axonal varicosities. Hernández et al. examined age-dependent variations in GABA and ACh incidence and segregation in rat SGC. These neurotransmitters were found more segregated in 2- and 4-week-old animals, with a caudal-rostral segregation gradient in 8- and 12-week-old animals. Data show that GABA and ACh incidence and segregation fluctuate throughout rats’ lives. The authors propose that early postnatal increase in GABA-ACh segregation increases GABA release, possibly exerting trophic effects.
